# Decoding the anomalies: a genome-based analysis of *Bacillus cereus* group strains closely related to *Bacillus anthracis*

**DOI:** 10.3389/fmicb.2025.1527049

**Published:** 2025-02-05

**Authors:** Thuto Gomolemo Magome, Marius Surleac, Ayesha Hassim, Cornelius Carlos Bezuidenhout, Henriette van Heerden, Kgaugelo Edward Lekota

**Affiliations:** ^1^Unit for Environmental Sciences and Management, Microbiology, North-West University, Potchefstroom, South Africa; ^2^The Research Institute of the University of Bucharest, Bucharest, Romania; ^3^National Institute for Infectious Diseases “Matei Balș”, Bucharest, Romania; ^4^Department of Veterinary Tropical Diseases, Faculty of Veterinary Science, University of Pretoria, Onderstepoort, South Africa

**Keywords:** *Bacillus cereus* group, *Bacillus anthracis*, whole genome sequencing, pan-genomics, average nucleotide identity

## Abstract

**Introduction:**

The *Bacillus cereus* group encompasses a complex group of closely related pathogenic and non-pathogenic bacterial species. Key members include *B*. *anthracis*, *B*. *cereus*, and *B*. *thuringiensis* organisms that, despite genetic proximity, diverge significantly in morphology and pathogenic potential. Taxonomic challenges persist due to inconsistent classification methods, particularly for *B*. *cereus* isolates that resemble *B*. *anthracis* in genetic clustering.

**Methods:**

This study investigated *B*. *cereus* group isolates from blood smears of animal carcasses in Kruger National Park, uncovering an unusual isolate with *B*. *cereus* features based on classical microbiological tests yet *B*. *anthracis*-like genomic similarities with an Average Nucleotide Identity (ANI) of ≥95%. Using comparative genomics, pan-genomics and whole genome Single Nucleotide Polymorphism (wgSNP) analysis, a total of 103 *B*. *cereus* group genomes were analyzed, including nine newly sequenced isolates from South Africa and a collection of isolates that showed some classification discrepancies, thus classified as “anomalous.”

**Results and discussion:**

Of the 36 strains identified as *B*. *anthracis* in GenBank, 26 clustered phylogenetically with the four confirmed *B*. *anthracis* isolates from South Africa and shared 99% ANI. Isolates with less than 99% ANI alignment to *B*. *anthracis* exhibited characteristics consistent with *B*. *cereus* and/or *B*. *thuringiensis*, possessing diverse genetic profiles, insertion elements, resistance genes, and virulence genes features, contrasting with the genetic uniformity of typical *B*. *anthracis*. The findings underscore a recurrent acquisition of mobile genetic elements within *B*. *cereus* and *B*. *thuringiensis*, a process infrequent in *B*. *anthracis*.

**Conclusion:**

This study highlights the pressing need for standardized taxonomic criteria in *B*. *cereus* group classification, especially as anomalous isolates emerge. This study supports the existing nomenclature framework which offers an effective solution for classifying species into genomospecies groups. We recommend isolates with ANI ≥99% to standard reference *B*. *anthracis* be designated as typical *B*. *anthracis* in GenBank to maintain taxonomic clarity and precision.

## 1 Introduction

The *Bacillus cereus sensu lato* (*s*. *l*) also referred as *B*. *cereus* group, is a complex cluster of Gram-positive, spore-forming, rod-shaped bacteria comprising both pathogenic and non-pathogenic species (Okinaka and Keim, 2016). Over 18 members have been classified as part of the *B*. *cereus* group; *B*. *albus*, *B. anthracis*, *B. cereus*, *B. cytotoxicus*, *B. luti*, *B. mobilis*, *B. mycoides*, *B. nitratireducens B. pacificus*, *B. paranthracis*, *B. paramycoides*, *B. proteolyticus*, *B. pseudomycoides*, *B. toyonensis*, *B. thuringiensis*, *B. tropicus*, *B. wiedmannii*, and *B. weihenstephanensis* (Carroll et al., 2020a,b). The most prominent pathogenic species include *B. anthracis*, *B. cereus*, and *B. thuringiensis*, each displaying unique phenotypic and virulence traits. For instance, *B. anthracis* presents as non-motile, encapsulated, non-hemolytic in sheep blood agar, and sensitive to both γ-phage and penicillin (WHO, 2008), while *B. cereus* and *B. thuringiensis* are hemolytic, motile, non-encapsulated, and exhibits γ-phage and penicillin resistance (Vilas-Bôas et al., 2007; Kolstø et al., 2009).

*Bacillus anthracis* is a zoonotic pathogen which causes anthrax disease (Turnbull, 2002). The disease primarily affects livestock, wildlife and humans and may present as cutaneous anthrax, acquired through contact with contaminated food or meat of animal with the disease, and inhalation anthrax, from breathing in airborne anthrax spores (WHO, 2008). Pathogenicity in *B. anthracis* is primarily attributed to plasmids pXO1 and pXO2 which harbor the anthrax toxin genes that include protective antigen (*pagA*), lethal factor (*lef*) and edema factor (*cya*) and the poly-γ-D-glutamic acid (PGA) capsule genes (*capABCDE*), respectively (Turnbull, 2002; Pena-Gonzalez et al., 2018). In contrast, *Bacillus cereus* acts as an opportunistic pathogen capable of causing gastrointestinal and non-gastrointestinal infections (Kotiranta et al., 2000; Bottone, 2010). Gastrointestinal disease may manifest as diarrheal (linked to toxins such as hemolysin BL, non-hemolytic enterotoxin, and cytotoxin K) or emetic illness (due to the cereulide toxin encoded by *cesABCDPTH*) (Schoeni and Wong, 2005; Owusu-Kwarteng et al., 2017; Ehling-Schulz et al., 2006). On the other hand, *Bacillus thuringiensis*, often employed as a natural insecticide which is not harmful to humans, contains insecticidal crystal protein genes (*cry* and/or *cyt*), which may lead to its misidentification as *B. cereus* in the absence of these genes (Kolstø et al., 2009).

Due to the clinical, agricultural, and economic importance of *B. cereus s.l*, especially the pathogenic species, substantial research has focused on their classification and taxonomy (Baldwin, 2020; Carroll et al., 2020a,b). The classification of these species was historically based on characteristics such as hemolytic activity, colony morphology, γ-phage and penicillin activity and the detection of virulence markers that were alleged to be species-specific (Rasko et al., 2005; Kamar et al., 2013; Ehling-Schulz et al., 2019). However, the emergence of exceptional genomes, such as *B. thuringiensis* isolates carrying *cry* genes that phylogenetically grouped with *B. anthracis* (Kolstø et al., 2009), along with atypical *B. cereus* and *B. cereus* biovar *anthracis* strains carrying the pBCXO1 and pBCXO2 plasmids, which contain anthrax virulence factors (Antonation et al., 2016; Baldwin, 2020), and the loss of virulence plasmids in *B. anthracis* isolates (Marston et al., 2005), suggested that the identification and classification of *B. cereus* group isolates should not rely solely on phenotypes and virulence factors (Baldwin, 2020; Brézillon et al., 2015). In some instances, molecular analysis targeting virulence determinants of *B. anthracis* revealed the presence of homologous PGA that synthesizes capsular genes (*capABCDE*) of *B. anthracis* in other *Bacillus* species (*pgsABCDE*) (Lekota et al., 2016; Lekota et al., 2018). Moreover, varying phenotypic characteristics by expressed bacterial isolates and/or limited genomic databases may have led to nomenclatural discrepancies observed in the *B. cereus* group (Afshinnekoo et al., 2015; Abdelli et al., 2023), which could have profound implications for clinical and public health settings (Carroll et al., 2020a,b; Carroll et al., 2022a,b,c). To date, the phenotypic characteristics (i.e., hemolysis activity, capsule presence/absence), plasmids and virulence factors still play a critical role in routine diagnosis and surveillance of potential disease outbreaks associated with pathogenic members of the *B. cereus* group (Mogaji et al., 2024).

Genetic techniques such as DNA–DNA hybridization (DDH) (Goris et al., 2007), multiple-locus variable-number tandem repeat analysis (MLVA) (Marston et al., 2006), amplified fragment length polymorphisms (Guinebretière et al., 2008), 16S RNA (Braun et al., 2021) and 23S RNA (Sacchi et al., 2002) were entirely instrumental methods used to differentiate members of the *B. cereus* group. The growing scheme of technology and rising novel species saw an increase in the application of more gene-specific target sequencing, such as single- and multi-locus sequence typing (SLST and MLST) methods, which became and remained some of the essential tools used for the identification of the *B. cereus* group members (Guinebretière et al., 2008). The pantoate-ß-alanine ligase (*panC*) emerged as a popular target locus used in SLST to assign members of the *B. cereus* group into seven distinct phylogenetic groups (group I-VII) based on sequence variations providing superior resolution compared to other traditional methods such the 16S RNA (Guinebretière et al., 2008). The seven-group assignment was later updated to an eight-group *panC* group assignment/genomospecies (Group I-VIII) with species names as representative names for some of the assigned groups (Carroll et al., 2022a,b,c). Group I contains species closely related to *B. pseudomycoides*. Group II (mosaicus/luti) includes certain strains that exhibit unique characteristics but are less commonly identified. Group III (mosaicus) is notably the most diverse group containing strains with emetic toxin production (cereulide), anthrax virulence genes production, and traditional *B. anthracis*, *B. cereus* and *B. thuringiensis* species closely related to traditional *B. anthracis*. Group IV (*B. cereus sensu stricto*) is a group that contains strains closely related to *B. cereus sensu stricto*, frequently identified in clinical and food-related sources. *Bacillus thuringiensis* species are also located in Group IV. Group V (*B. toyonensis*) contains strains that have been less frequently characterized but are part of the broader *Bacillus cereus* group. Group VI (*B. mycoides/paramycoides*) primarily consists of psychrotolerant species, indicating their ability to grow at lower temperatures (Liu et al., 2018). Group VII (*B. cytotoxicus*) contains specific strains with cytotoxic effects related to *cytotoxin K-1* (Fagerlund et al., 2004) and Group VIII (*B. mycoides*), which contains species closely related to *B. mycoides.*

The study of Liu et al. (2015) applied an integrated approach utilizing molecular techniques, including digital DNA–DNA hybridization (dDDH) with a ≥70% similarity threshold (Goris et al., 2007), MLST, and 16S RNA analysis. This approach clustered 224 genomes of *B. cereus s.l* into 30 clusters. Notably, dDDH analysis revealed that 20 genomes initially classified as *B. cereus* or *B. thuringiensis* exhibited unique genomic characteristics and were subsequently identified as *B. anthracis*, referred to as ‘anomalous *B. anthracis*’ to distinguish them from traditional *B. anthracis* isolates (Liu et al., 2015). In a separate study, 2,231 *B. cereus* group genomes were analyzed using Fast ANI, which found that approximately 66.2% of the genomes belong to several genomospecies at an ANI threshold of 95% (Carroll et al., 2020a,b). Furthermore, additional anomalous *B. cereus* group isolates were identified, including isolates with phenotypic features typical of *B. anthracis*, *B. cereus*, and *B. thuringiensis*, which exhibited an ANI ≥ 95% with canonical *B. anthracis*, yet lacked anthrax-specific virulence genes. This work also proposed a nomenclatural framework which integrated virulence detection, MLST, ANI and *panC* group assignment for the *B. cereus* group to harmonize phenotypic and genomic classification, aiming to reduce misclassification and misinterpretation of species in clinical and industrial settings, ultimately addressing public health implications (Carroll et al., 2020a,b).

The estimated pan-genome of the *B. cereus* group encompasses approximately 60,000 genes, with around 600 core genes shared by 99% of analyzed strains (Bazinet, 2017). This estimate, however, is likely evolving with the classification of new members and the availability of additional genomes. Pan-genome analysis has been instrumental in distinguishing essential core and accessory genes, revealing the functional versatility of the *B. cereus* group and the unique adaptive capacities of individual strains (Kim et al., 2017). While pan-genome analysis illuminates the broader genetic landscape, providing insights into environmental adaptability and metabolic functionality, whole-genome single nucleotide polymorphism (wgSNP) analysis offers a high-resolution for delineating differentiating genetic diversity within closely related strains (Bogaerts et al., 2023). Single nucleotide polymorphisms are evolutionarily stable markers that contribute to elucidating deep phylogenetic relationships among global strains (Pearson et al., 2004; Girault et al., 2014; Lekota et al., 2024). Given the monomorphic nature of *B. anthracis*, wgSNP analysis has proven valuable in differentiating traditional *B. anthracis* strains from other closely related *B. cereus* group members.

This study investigated the genomic cohesion among “anomalous” *B. cereus* group isolates, prompted by the identification of an isolate with phenotypic features consistent with *B. cereus* yet a phylogenetic profile aligning with typical *B. anthracis*. A genome-based comparative approach was employed, encompassing pan-genomic and wgSNP analyses of previously reported “anomalous” *B. cereus* group strains from the studies of Liu et al. (2015) and Carroll et al. (2020a,b), as well as newly sequenced isolates displaying typical *B. anthracis* and *B. cereus* phenotypes. The isolates investigated in this study were obtained from archival animal blood smears collected in anthrax endemic regions of Kruger National Park, South Africa, as part of their anthrax outbreak surveillance program. The isolates in this study were initially screened for anthrax virulence markers (*pagA*, *lef*, and *capB*) and the chromosomal marker Ba-1, with phenotypic characteristics of the isolated recorded as detailed in Ochai et al. (2024). The collaborative work of the surveillance program is put in place to monitor anthrax outbreaks caused by traditional *B. anthracis* and/or any other potential isolates that may cause anthrax disease.

## 2 Materials and methods

### 2.1 Sample collection and screening

Bacterial cultures of samples collected between 2012 and 2015 (Table 1), were isolated from blood smears obtained from animal carcasses from the anthrax endemic regions of Kruger National Park (KNP) as described by Ochai et al. (2024). Briefly, blood smears on microscope slides were sterilely scrapped into 1.5 mL centrifuge tubes. Two hundred microliters of phosphate-buffered saline (PBS; Thermo Scientific, MA, United States) was added into the tube and half of the aliquot was spread plated on 5% sheep blood agar and incubated overnight at 37°C. All isolates with different colony morphologies were treated as different isolates and were sub-cultured on 5% sheep blood agar to obtain pure colonies. All the isolates were primarily subjected to classical methods that included microscopy, morphology, motility, hemolysin activity, γ-phage and penicillin sensitivity tests as described by the World Health Organization (WHO, 2008). The isolates were screened for the presence/absence of anthrax-toxin markers (*pagA*, *lef*, *capB*) and the *B. anthracis* chromosomal marker Ba-1 using SYBR green as prescribed by WHO (2008) and Taqman probe qPCR-based method as prescribed by Zincke et al. (2020). This was done in order to investigate any other *Bacillus* isolates which may carry anthrax virulence genes. The results are summarized in Supplementary Table S1, extracted from the study of Ochai et al. (2024).

**Table 1:** *Bacillus anthracis* and *B. cereus* isolates from South Africa were cultured from blood smears from animal carcasses in the Kruger National Park, South Africa.

Strain	Genus	Animal source	Genus/species	Ranger section
AX2012-121	*Bacillus*	Wildebeest	*Connochaetes*	Mooiplaas
AX2013-496	*Bacillus*	Rhinoceros	*Diceros bicornis*	Houtboschrand
AX2014-912	*Bacillus*	Impala	*Aepyceros melampus*	Lower Sabie
AX2014-949	*Bacillus*	Rhinoceros	*Diceros bicornis*	Houtboschrand
AX2016-1771Ac	*Bacillus*	Zebra	*Equus quagga*	Pafuri
AX2015-1136	*Bacillus*	Impala	*Aepyceros melampus*	Pafuri
AX2015-1152	*Bacillus*	Nyala	*Tragelaphus angasii*	Pafuri
AX2015-1270	*Bacillus*	Zebra	*Equus quagga*	Pafuri
AX2015-1277A	*Bacillus*	African Buffalo	*Syncerus caffer*	Mooiplaas

### 2.2 Genomic extractions and sequencing

Genomic DNA of the isolates (*n* = 9) was extracted from overnight pure cultures using the Pure link Genomic DNA kit (Thermo Fisher Scientific, United States) following the manufacturer’s protocol. The concentration and quality of the DNA were determined using the Qubit 2.0 fluorometer (Thermofisher-Scientific, United States). The DNA was sent to the Agricultural Research Council-Biotechnology Platform (ARC-BTP) for whole genome sequencing. Sequence libraries of the isolates were constructed using the MGIEasy FS DNA Prep Kit (BGI, China) according to the manufacturer’s protocol. The prepared libraries were sequenced using the BGI MGISEQ-2000 platform (BGI Shenzhen, China) using the paired-end 2 × 150 bp to generate reads. Four genomic sequences (AX2015-1136, AX2015-1152, AX2015-1270, and AX2015-1277A) were described and published in our previous study (Magome et al., 2024). For this study, we included these four isolates as they form part of an anthrax surveillance study in KNP from the same culture collection of the *B. cereus* group.

### 2.3 Genome assembly and annotation

*De novo* sequencing assembly, adapter trimming and polishing were performed using Shovill v4.6.0 (Seemann et al., 2020). Transeq from EMBOSS was used for the translation of nucleotide sequences into amino acids (Rice et al., 2000). Diamond software was used to compare the contig sequences against the protein databases by BlastX (the following command option was used: diamond blastx --max-target-seqs 0 --more-sensitive --id 70 -p 8 --subject-cover 90) (Buchfink et al., 2015; Buchfink et al., 2023). In-house AWK, Python and Excel scripts were further used to filter the resulting data. The annotation of the selected isolates was first performed with Prokka v1.14 using the command option: prokka --cpus 8 --gcode 11 --rnammer --compliant --center XXX (Seemann, 2014), an updated annotation tool Bakta (Schwengers et al., 2021) was then used with the following command argument: bakta --db db --verbose --threads 8. The resulting output from annotations was further used as input for Roary 3.13.0 (the following options have been used: “*-g 80000 -e --mafft -p 8 -r -qc -r -z -f*”) (Page et al., 2015). Gene ontology classification of the pangenome data, was performed using DeepNOG (Feldbauer et al., 2020) and COG Classifier tools (Shimoyama, 2022). Whole genome SNP analysis was performed using Snippy v4.6.0^1^ (Seemann, 2015). The clean alignment of the sequences was subjected to bootstrap =100 phylogenetic tree built with RaXML (Stamatakis, 2014).

### 2.4 Genome identification and phylogenetic placement

The sequenced genomes were first identified using the Pub-MLST species-ID search tool^2^ (Jolley et al., 2018) and the Genome Taxonomy Database (GTDB) v1.7.0 which incorporates the Fast Average Nucleotide Identity (ANI) on KBase app (Arkin et al., 2018). Pan-genomic placement together with ANI comparison using the sequenced genomes (*n* = 9) and the 18 recognized members of the *B. cereus* group obtained from GenBank was constructed using the integrated prokaryotes genome and pan-genome analysis web services (IPGA) v1.09^3^ (Liu et al., 2022). A comprehensive comparative genomics analysis was conducted on 88 anomalous genomes of the *B. cereus* group, which were retrieved from GenBank and classified into three major species: *B. anthracis* (*n* = 36), *B. cereus* (*n* = 37), and *B. thuringiensis* (*n* = 15). This analysis included five *B. cereus* biovar *anthracis* strains (CAM, CAR, CI, DRC, and UFBc0001) sourced from animal samples in Cameroon, the Central African Republic, Côte d’Ivoire, and the Democratic Republic of the Congo. These strains have reportedly caused anthrax-like diseases in their hosts (Antonation et al., 2016; Baldwin, 2020). Additionally, the atypical *B. cereus* strain BC-AK, isolated from a kangaroo in China, was included, along with nine South African isolates from this study (Supplementary Tables S2, S3). In total, this analysis examined 103 genomes. Moreover, further description of the genomes was conducted using BTyper3 (Carroll et al., 2020a,b) that involved *panC* group assignment, PubMLST genome identification, GTDB identification and ANI comparison within the IPGA (Liu et al., 2022). The pan-genome trees were based on gene presence/absence and were visualized using tvBOT incorporated in the web-based tool ChiPlot^4^ (Xie et al., 2023).

### 2.5 Identification of mobile elements, virulence factors, and resistance genes

Various nucleotide/protein sequence databases, such as comprehensive antibiotic resistance database (CARD) (Alcock et al., 2023), ResFinder (Feldgarden et al., 2019), BacMet (Pal et al., 2014), BacAnt (Hua et al., 2021), MobileElementFinder (Johansson et al., 2021), PAIDB (Yoon et al., 2015), Iceberg2 (Liu M. et al., 2019; Liu B. et al., 2019) were used for mobile genetic elements and resistance gene predictions. Specifically, antibiotic resistance determinants were identified in each assembled genome using the ResFinder [−db ResFinder] (Feldgarden et al., 2019) with the minimum identity and coverage thresholds of 75 (−minid 75) and 50% (−mincov 50), respectively. Virulence factors in the sequenced genomes were mined using the Virulence Factor Database [−db vfdb] (Chen et al., 2016; Liu M. et al., 2019; Liu B. et al., 2019), using minimum identity and coverage thresholds of 75 (−minid 75) and 50% (−mincov 50), respectively. Further mining and annotation of antibiotic resistance genes (ARG), integrons and transposable elements was conducted using the BacAnt web-based tool (Hua et al., 2021), whereas BacMet was used to mine for metal resistance genes (Pal et al., 2014).

## 3 Results

### 3.1 Phenotypic and molecular characteristics

Microscopic examination of the nine bacterial isolates revealed that all were Gram-positive rod-shaped cells. Notably, four isolates (AX2015-1136, AX2015-1152, AX2015-1270, and AX2015-1277A) displayed long rod chains, while the remaining five isolates (AX2012-121, AX2013-496, AX2014-912, AX2014-949, and AX2016-1771Ac) were characterized by shorter rod-shaped cells, presumptively identified as *B. cereus*. Among these, the latter five were β-hemolytic, motile, and showed γ-phage and penicillin resistance. Conversely, the four isolates previously identified as *B. anthracis* (AX2015-1136, AX2015-1152, AX2015-1270, and AX2015-1277A) were non-hemolytic, γ-phage and penicillin sensitive (Ochai et al., 2024). Molecular screening of these isolates for anthrax virulence genes indicated the presence of *pagA*, *lef*, and the chromosomal marker Ba-1 in *B. anthracis* isolates. The other five isolates, AX2012-121, AX2013-496, AX2014-912, AX2014-949, and AX2016-1771Ac, tested positive for the *lef* gene. The *B. cereus* isolate AX2014-912 tested positive for anthrax chromosomal marker Ba-1, while isolate AX2016-1771Ac amplified for the *capB*, *lef*, and *pagA* marker (Supplementary Table S1).

### 3.2 Genome metrics and identification of the *Bacillus cereus* group isolates

The genome features of the sequenced isolates are presented in Table 2. Quality assessment of the assembled genomes showed an average of 99.43% completeness for all the genomes (*n* = 9). The genomes were initially identified using Pub-MLST and GTDB-tk v1.7.0, which incorporates Fast average nucleotide identity (ANI) by matching the query genomes against the closest reference strains incorporated in the database. The isolates AX2015-1136, AX2015-1152, AX2015-1270, and AX2015-1277A were identified as *B. anthracis* based on PubMLST species identifier and shared an ANI of ≥99% score when matched with the *B. anthracis* Vollum reference isolate (GCA_000007825.1) based on GTDB-tk v1.7.0. The isolates AX2012-121, AX2013-496, AX2014-912, and AX2014-949 were classified as *B. cereus*, based on PubMLST species identifier and each shared ≥98 ANI score when matched with *B. cereus* ATCC 14579 (GCA_000008725.1) using GTDB-tk v1.7.0. The genome size of the *B. cereus* genomes ranged from 5.34 Mb to 5.52 Mb, with a GC content ranging from 35.0 to 35.2%. Isolate AX2016-1771Ac was identified as *B. cereus* on the PubMLST species identifier. However, based on GTDB-tk v1.7.0, the closest reference genome to isolate AX2016-1771Ac was *B. anthracis* Vollum (GCA_000007825.1). The two genomes shared ≥97 ANI, which is well above the 95% ANI threshold typically used for prokaryotic species delineation (Jain et al., 2018).

**Table 2:** Genome features of the nine South African sequenced *Bacillus cereus* group isolates.

Isolate	PubMLST	GTDB-tk and BTyper3-PubMLST	Contigs	Largest contig	Genome size (bp)	N50	G + C Content %	Coding sequences	RNAs
AX2015-1136	*B. anthracis*	*B. anthracis*	72	548,990	5,359,201	157,399	35.1	5,943	77
AX2015-1152	*B. anthracis*	*B. anthracis*	76	641,194	5,459,155	186,582	35.1	5,861	58
AX2015-1270	*B. anthracis*	*B. anthracis*	59	450,267	5,445,999	221,866	35.1	5,847	49
AX2015-1277A	*B. anthracis*	*B. anthracis*	43	451,964	5,451,556	538,273	35.1	5,848	52
AX2012-121	*B. cereus*	*B. cereus*	90	833,493	5,524,290	194,899	35.0	5,598	58
AX2013-496	*B. cereus*	*B. cereus*	143	443,994	5,489,039	85,188	35.1	5,617	77
AX2014-912	*B. cereus*	*B. cereus*	126	813,864	5,466,335	113,038	35.1	5,593	69
AX2014-949	*B. cereus*	*B. cereus*	98	430,535	5,431,140	211,170	35.2	5,494	74
AX2016-1771Ac	*B. cereus*	*B. anthracis*	44	616,581	5,338,489	304,668	35.1	5,568	54

**Figure 1:**
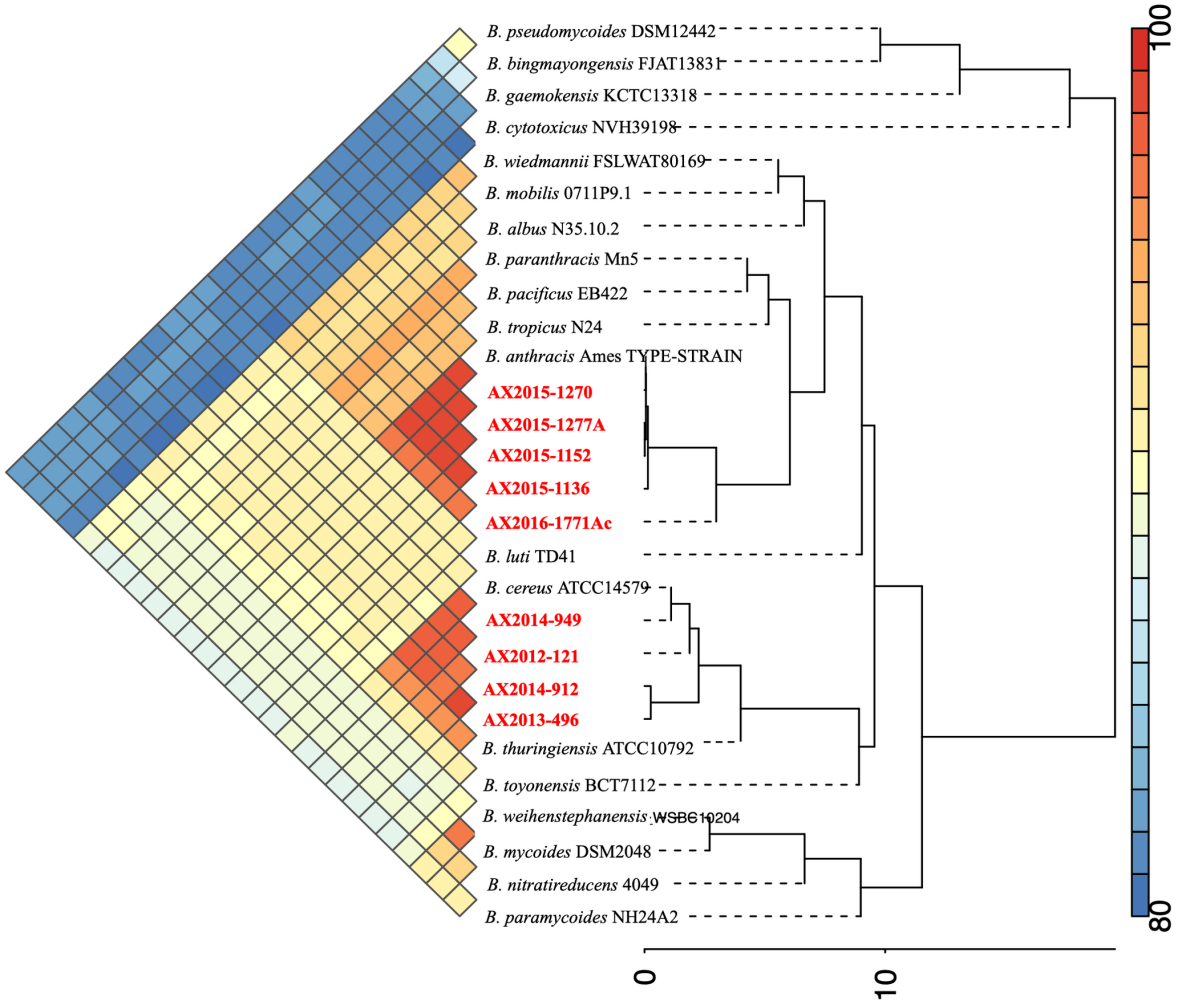
Phylogenetic placement and Average Nucleotide identity comparison using the 18 recognized members of the *B. cereus* group obtained from Carroll et al. (2020a,b), including the South African genomes (*n* = 9) from this study (marked in red).

The genome size of AX2016-1771Ac was 5.33 Mb with a GC content of 35.1% (Table 2). Phylogenetic placement using pan-genome and ANI analysis of the 18 recognized *B. cereus* group isolates in IPGA v1.09 (Figure 1) showed that isolates identified as *B. cereus* (AX2012-121, AX2013-496, AX2014-912, and AX2014-949) grouped with *B. cereus* ATCC 14579. The *B. anthracis* genomes (AX2015-1136, AX2015-1152, AX2015-1270, and AX2015-1277A) grouped more closely with *B. anthracis* Ames TYPE-STRAIN (GCA_000007845.1). However, isolate AX2016-1771Ac grouped closely with the *B. anthracis* isolates than with *B. cereus* isolates (Figure 1).

### 3.3 Phylogenetic placement of the *Bacillus cereus* group genomes using various databases

**Figure 2:**
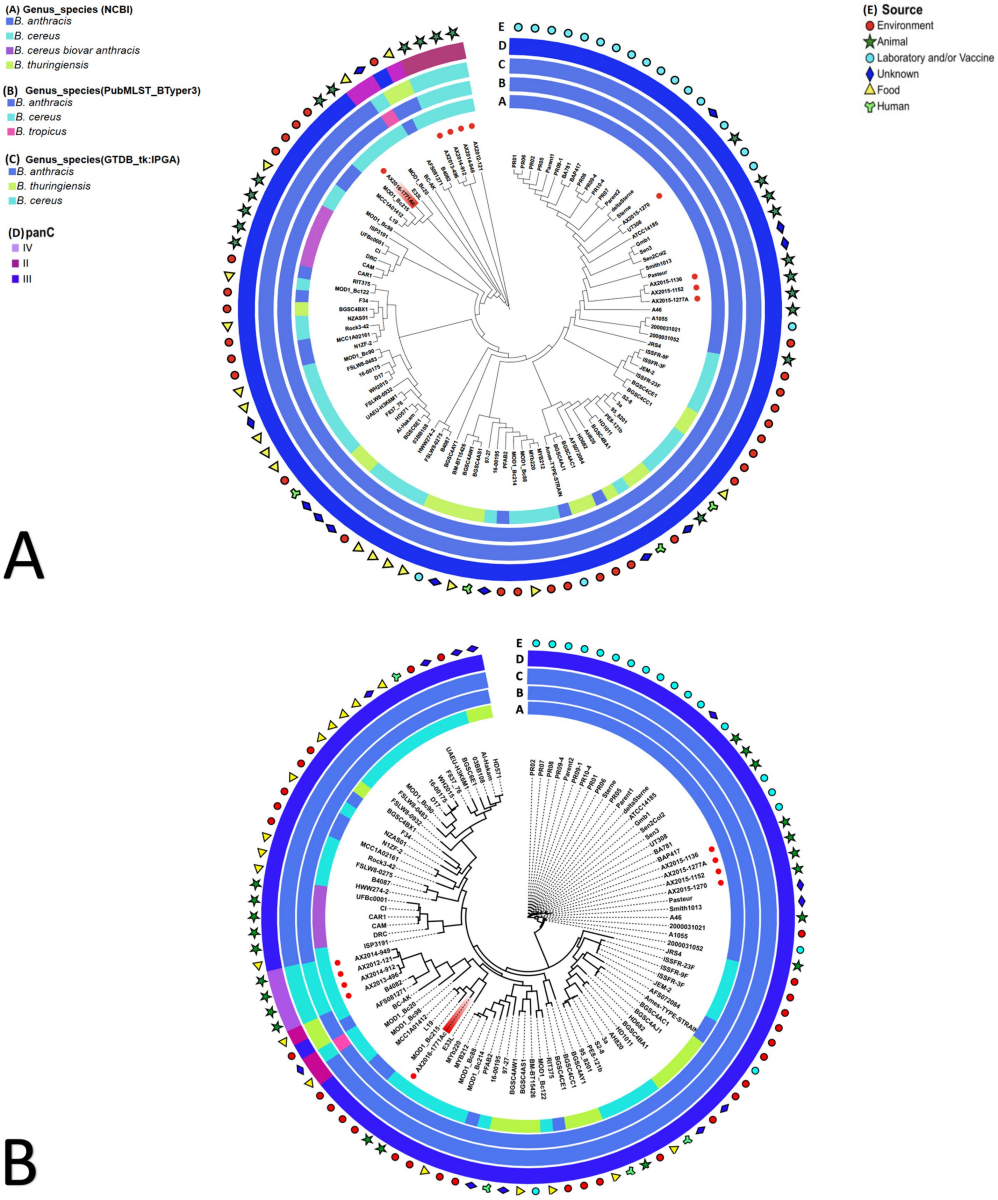
**(A)** Pan-genome and **(B)** whole genome single nucleotide polymorphism (wgSNP) analysis of 103 *Bacillus cereus* group genomes analyzed. The red dots in the inner circle indicate the South African isolates from this study. The AX2016-1771Ac highlighted in red. The rings surrounding the phylogenetic tree represent the following: (A) current genus species assignment as submitted on GenBank database; (B) BTyper3 PubMLST genome species assignment; (C) GTDB-tk-IPGA species assignment; (D) *panC* group assignment; (E) general source from which samples were obtained from.

The pan-genome and wgSNP analysis revealed that four isolates (AX2015-1136, AX2015-1152, AX2015-1270, and AX2015-1277A) from South Africa identified as *B. anthracis* based on phenotypic characteristics, GTDB-tk and PubMLST classification, clustered with 26 additional genomes identified as *B. anthracis* on GenBank (PR01, PR02, PR05, PR06, PR07, PR08, PR09-1, PR09-4, PR10-4, Parent1, Parent2, Sterne, deltaSterne, BA_V770-NP1-R-ATCC 14185, Gmb1, Sen3, Sen2Col2, UT308, BAP417, BA781, Smith1013, Pasteur, A46, A1055, 2000031021, and 2000031052). This collective group, totalling 30 genomes, was consistently classified as *B. anthracis* based on GTDB-tk and BTyper3-PubMLST analysis and clustered in the mosaicus *panC* group III, sharing a ≥ 99% ANI (Figure 2; Supplementary Tables S2–S5). Given the genetic monomorphism characteristic of traditional *B. anthracis* strains, the wgSNP analysis (Figure 2B) supports the inclusion of these isolates within the *B. anthracis* lineage. Thus the 30 genomes are proposed to be accepted as part of the traditional or typical *B. anthracis.* This group of strains displayed diverse isolation sources, including laboratory/vaccine strains (*n* = 17), environment-soil (*n* = 1), animals (*n* = 9), and unknown source type (*n* = 3) from locations including the United States of America (USA), Senegal, Gambia, Pakistan and South Africa (Figure 2).

Ten of the genomes classified on GenBank as *B. anthracis* (Ames TYPE-STRAIN, AFS072084, PFAB2, L19, MCC1A01412, AFS081271, MCC1A02161, N1ZF-2, RIT375, and F34) did not co-cluster with the previously mentioned 30 *B. anthracis* genomes based on pan-genome and wgSNP analyses (Figure 2). Furthermore, ANI analysis showed that the 10 genomes shared ≤98 ANI identity with typical *B. anthracis* genomes (AX2015-1136, AX2015-1152, AX2015-1270, and AX2015-1277A) (Supplementary Table S5). These genomes originated from diverse sources, such as hot spring (India, strain PFAB2), salt lake (Algeria, strain F34), sediment (South China sea: strains MCCC1A02161, MCC1A01412, N1ZF-2, and L19), soybean plant (strain AFS081271), corn plant (strain AFS072084), stem tissue of a *Chamaecostus cuspidatus* plant (Puerto Rico, strain RIT375) and laboratory/vaccine (Ames TYPE-STRAIN). Despite being classified as *B. anthracis* under BTyper3-PubMLST and as *panC* group III within the mosaicus group, one genome (AFS081271) was classified as *B. thuringiensis* by GTDB-tk in IPGA, aligning with isolate B4082 classified as *B. cereus* in GenBank isolated from a food source (pea soup in the Netherlands) (Figure 2). The genomes AFS081271 and B4082 were classified as *B. thuringiensis* based on GTDB-tk in IPGA v1.09 and classified as *B. anthracis* based on Btyper3 PubMLST belonging to *panC* group II of mosaicus/luti (strain B4082) and in *panC* group III of the mosaicus group (strain AFS081271) (Figure 2). The genomes AFS081271 and B4082 shared a ≥ 99% ANI when compared against each other, suggesting they are the same species. Moreover they clustered with four South African isolates (AX2012-121, AX2013-496, AX2014-912, and AX2014-949) identified as *B. cereus* under GTDB-tk and PubMLST (Figure 2).

The four *B. cereus* genomes, isolated from animal blood smears in South Africa, grouped under *panC* group IV in the *B. cereus sensu stricto* group based on Btyper3, showing 97–98% ANI among themselves and ≤ 91% ANI compared to the GenBank *B. cereus* genomes (Figure 2; Supplementary Tables S4, S5). Thirty-five GenBank *B. cereus* genomes were identified as *panC* group III of the mosaicus group and two GenBank *B. cereus* genomes (B4082 and MOD1_Bc20), isolated from food sources were identified as *panC* group II of the mosaicus/luti group, however they were classified as *B. anthracis* in BTyper3 (Figure 2). The results above highlight the discrepancies that exist within the various genomic identification databases which further complicate taxonomic classification. Additionally, an anomalous AX2016-1771Ac isolate, originating from a zebra blood smear, sequenced in this study, grouped with *B. cereus* (E33L and MOD1_Bc215) and *B. anthracis* (L19 and MCC1A01412) genomes using pan-genome and wgSNP analysis (Figure 2). These isolates, identified as *B. anthracis* under GTDB-tk and BTyper3-PubMLST, aligned with *panC* group III in the mosaicus group. The anomalous isolate AX2016-1771Ac shared 97–98% ANI with similar genomes from the South China Sea (strains L19 and MCC1A01412), United States baby wipes (MOD1_Bc215), and a Namibian zebra (strain E33L). Two *B. thuringiensis* strains (HD571 and Al-Hakam) that form part of the anomalous clustered with *B. cereus* strains BGSC6E1, 03BB108, F837_76, and UAEU-H3K6M1 as *panC* group III species of the mosaicus group (Figure 2). GenBank classified *B. cereus* strains (FLSW8-0483, WH2015, D17, and MOD1_Bc90) isolated from food in the United States and strain 16-00175 from France formed part of this group. Meanwhile, the *B. anthracis* Ames TYPE-STRAIN and AFS072084 genomes formed close associations with *B. thuringiensis* strains (BGSC4AJ1 from Mexico and BGSC4AC1 from India), with a notable ≥99% ANI score shared between *B. anthracis* Ames TYPE-STRAIN and *B. thuringiensis* BGSC4AJ1 (Supplementary Table S5).

The anomalous *B. cereus* ISP3191 genome isolated from food source, clustered closely with the *B. cereus* biovar *anthracis* strains (CAM, CAR, CI, DRC, and UFBc0001) isolated from animals (gorilla, chimpanzees, goat, and colobus monkey) in Western Africa, sharing ≥99% ANI. This cluster displayed a 97% ANI similarity with 82 other GenBank genomes, including the four typical *B. anthracis* strains (AX2015-1136, AX2015-1152, AX2015-1270, and AX2015-1277A) from this study. A subset of anomalous isolates (AFS081271, L19, B4082, MOD1_Bc20, MOD1_Bc98, and MOD1_Bc215) exhibited ≤96% ANI with *B. cereus* biovar *anthracis* isolates. Moreover, the *B. cereus* (AX2012-121, AX2013-496, AX2014-912, and AX2014-949) isolates from this study, shared <91% ANI with *B. cereus* biovar *anthracis.* The AX2015-1771Ac isolate shared ≥96% ANI score with *B. cereus* biovar *anthracis* isolates. However, AX2015-1771Ac shared <94% ANI with atypical *B. cereus* strain BC-AK. These results indicate that *B. cereus* group isolates may belong to different species clusters when a threshold ≥95% is adopted for all species in the same group.

### 3.4 Comparative annotation and functional analysis of *Bacillus cereus* group genomes

**Figure 3:**
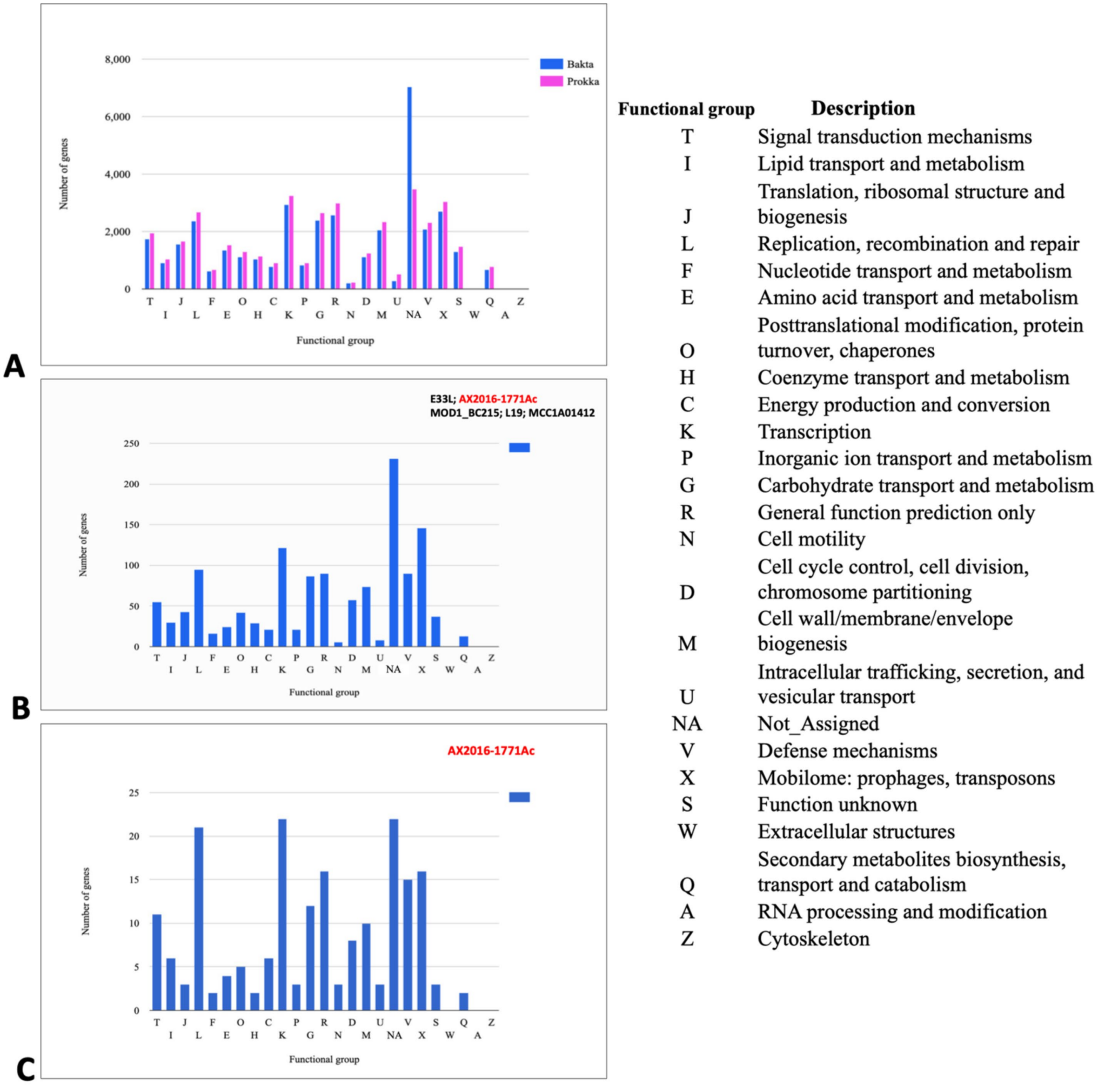
Cluster of orthologous groups (COGs) analysis indicating annotated genes in different functional groups **(A)** COG gene count comparison of the two annotation tools Prokka (pink) and Bakta (blue). **(B)** COG shared between the branch containing the five genomes *B. cereus* (AX2020-1771Ac, E33L, and MOD1_BC215) and *B. anthracis* (L19 and MCC1A01412) based on Bakta annotation. **(C)** COG corresponds to the set of genes that are unique to the anomalous sequenced isolate AX2016-1771Ac sequenced in this study. Each bar height corresponds to the total number of genes in the compartment that were assigned to the COG functional group. NA (Not Assigned compartment represents the genes that could not be assigned in a functional compartment).

To predict gene content and perform pan-genomic analysis, 103 genomes within the *Bacillus cereus* group were annotated using two distinct tools: Prokka and Bakta. Initially, the annotation based on Prokka identified a total of 38,079 genes, with 26,173 (68. 73%) classified as hypothetical proteins. In contrast, Bakta annotation assigned 37,678 genes, only 5,391 (14.31%) were predicted to be hypothetical proteins. This suggests that Bakta may offer improved functional predictions for gene products within the *B. cereus* group, potentially reducing ambiguity around hypothetical proteins (Figure 3).

In the Bakta annotation, 1,338 cloud genes were solely found in five anomalous isolates AX2016-1771Ac, *B. anthracis* strains (L19 and MCC1A01412) as well as *B. cereus* strains (E33L and MOD1_Bc215). Within this subset, 271 of these genes were assigned as hypothetical proteins. Additionally, 14 genes with distinct functional roles were identified exclusively in these five isolates. These genes included transport-related proteins (e.g., ABC transporter permease), structural proteins (e.g., spore coat protein), regulatory proteins (e.g., transcriptional regulators such as LysR and YdeE), metabolic enzymes (e.g., putative ubiquinone/menaquinone methyltransferase and NAD-dependent epimerase/dehydratase family protein), and other specific proteins (e.g., FAD-dependent oxidoreductase and DUF-domain proteins like DUF998, DUF952, DUF4430, and DUF3380). These unique genes may indicate specialized functionalities within these isolates. Functional categorization based on the cluster of orthologous groups (COG) analysis revealed that from the 1,338 clouds genes of the five compared strains, 146 genes (10.9%) were associated with mobilomes, including prophages and transposons functional group (X), followed by the transcription functional group (K) with 122 (9.1%) gene count, and the replication, recombination and repair functional group (L) with 95 (7.1%) gene counts (Figure 3B; Supplementary Table S6). Notably, the AX2016-1771Ac isolate contained 195 unique genes, which were not detected in other *B. cereus* group isolates, based on the COGs (Figure 3C).

### 3.5 Antibiotic resistance genes in *Bacillus cereus* group

**Figure 4:**
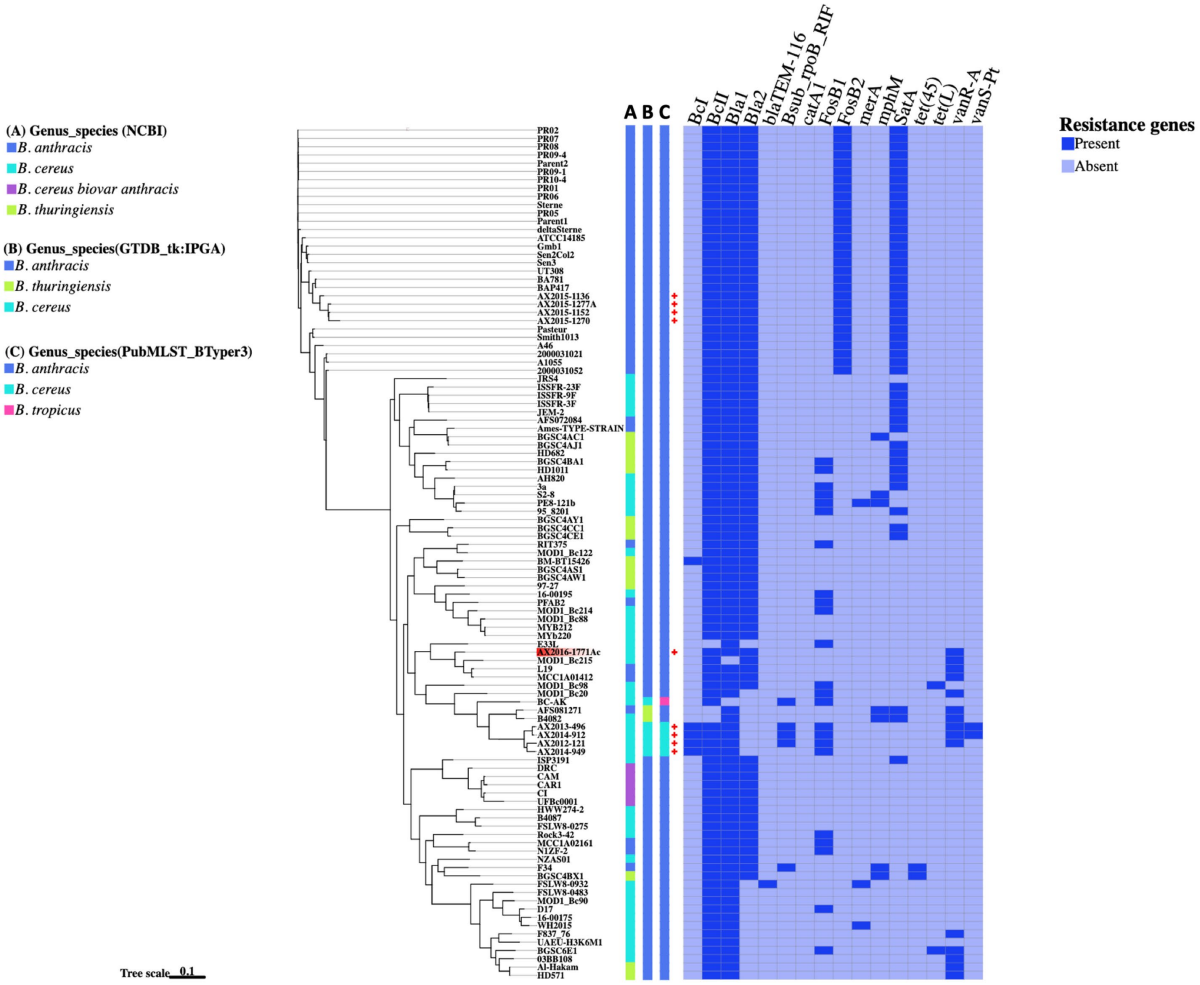
The presence and absence of resistance genes comparison between the *Bacillus* genomes. The phylogenetic tree is based on the wgSNP analysis of the 103 *Bacillus* genomes. The South African genomes from this study are marked with red crosses. A–C Blocks represents names assigned to the genomes on different genome identification databases: (A) Current genus species assigned to the genomes on NCBI; (B) Genus species assigned by the Genome taxonomy database in IPGA v1.09 (GTDB-tk); (C) Genus species assigned by the PubMSLT in BTyper3.

A total of 16 antibiotic resistance genes (ARGs) were distributed across 103 *Bacillus cereus* group genomes analyzed in this study (Figure 4). The most abundant ARGs identified across all genomes (*n* = 103) were the beta-lactamase genes *Bla1* (96%), *BcII* (95%), and *Bla2* (78%). A distinct class A beta-lactamase gene, *BcI*, was exclusively detected in four *B. cereus* genomes as (AX2012-121, AX2013-496, AX2014-912, and AX2014-949) and the anomalous GenBank classified *B. thuringiensis* strain BM-BT15426. Additionally, the beta-lactamase gene, *Bla_TEM-116_* was solely detected in the anomalous *B. cereus* strain FSLW8-0932, which was isolated from a food source in the United States. Another resistant gene, *satA* (encoding streptothricin N-acetyltransferase) was found in 48 genomes, but was absent in the *B. cereus sensu stricto* isolates (AX2012-121, AX2013-496, AX2014-912, and AX2014-949). The Fosfomycin resistance gene *FosB2* was identified in 30 genomes, all suggested in this study to represent traditional/typical *B. anthracis* with high genomic similarity (≥99 ANI) (Figure 2). Notably, the 30 *B. anthracis* isolates, exhibited a consistent ARG profile that included *BcII*, *Bla1*, *Bla2*, *FosB2*, and *satA* (Figure 4), a typical profile for *B. anthracis* isolates.

The fosfomycin gene *FosB1* was detected in 23 anomalous genomes made up of 12 *B. cereus* isolates (16-00195, 3a, 95_8201, D17, E33L, MOD1_Bc20, MOD1_Bc214, MOD1_Bc98, PE8-121b, Rock3-42, S2-8, and BGSC6E1), four *B. anthracis* isolates (RIT375, PFAB2, NIZF-2, and MCC1A02161), two *B. thuringiensis* isolates (HD10111 and BGSC4BA1), including the atypical *B. cereus* BC-AK strain and the four *B. cereus sensu stricto* isolates (AX2012-121, AX2013-496, AX2014-912, and AX2014-949) from this study (Figure 4). Furthermore, the vancomycin resistance gene, *vanR-A*, was detected in 15 genomes that included *B. cereus* classified genomes (AX2012-121, AX2013-496, AX2014-912, AX2016-1771AC, 03BB108, B4082, F837_76, MOD1_Bc20, MOD1_Bc215, and BGSC6E1), *B. anthracis* genomes (AFS081271, MCC1A01412, and L19) and *B. thuringiensis* (HD571 and Al-Hakam). The gene *rpoB* known to confer resistance to rifampicin was detected in the South African *B. cereus* strains (AX2012-121, AX2013-496, and AX2014-912), the atypical *B. cereus* BC-AK strain associated with anthrax-like disease, as well as in the anomalous *B. anthracis* F34 strain isolated from Algeria. The *vanS-Pt* gene that is associated with ruminant microbiota in *Paeniballicus* strains (Guardabassi et al., 2005) and vancomycin resistance was only detected in two *B. cereus* genomes AX2013-496 and AX2014-912 sequenced in this study. The tetracycline efflux pump *tet(45)* was detected in anomalous *B. anthracis* F34 and *B. thuringiensis* BGSC4BX1 isolates. The tetracycline efflux pump *tet(L)* was detected in the anomalous *B. cereus* isolates BGSC6E1 and MOD1_Bc98. The sequenced typical *B. cereus* isolates AX2013-496 and AX2014-912 each presented seven ARGs namely the *BcI*, *BcII*, *Bla1*, *rpoB*, *FosB1*, *vanR-A*, and *vanS-Pt.* The *B. cereus* AX2012-121 strain contained six ARGs namely the *BcI*, *BcII*, *Bla1*, *rpoB*, *FosB1*, and *vanR-A*. The *B. cereus* AX2014-949 strain contained only four ARGs, which were identified as *BcI*, *BcII*, *Bla1*, and *FosB1.* The anomalous *B. cereus* strain AX2016-1771Ac also contained four ARGs namely *BcII*, *Bla1*, *Bla2*, and *vanR-A. Bacillus cereus* and *B. thuringiensis* isolates indicated a potential to carry varying resistance gene profiles from the typical *B. anthracis* isolates.

### 3.6 Virulence factors in *Bacillus cereus* group

**Figure 5:**
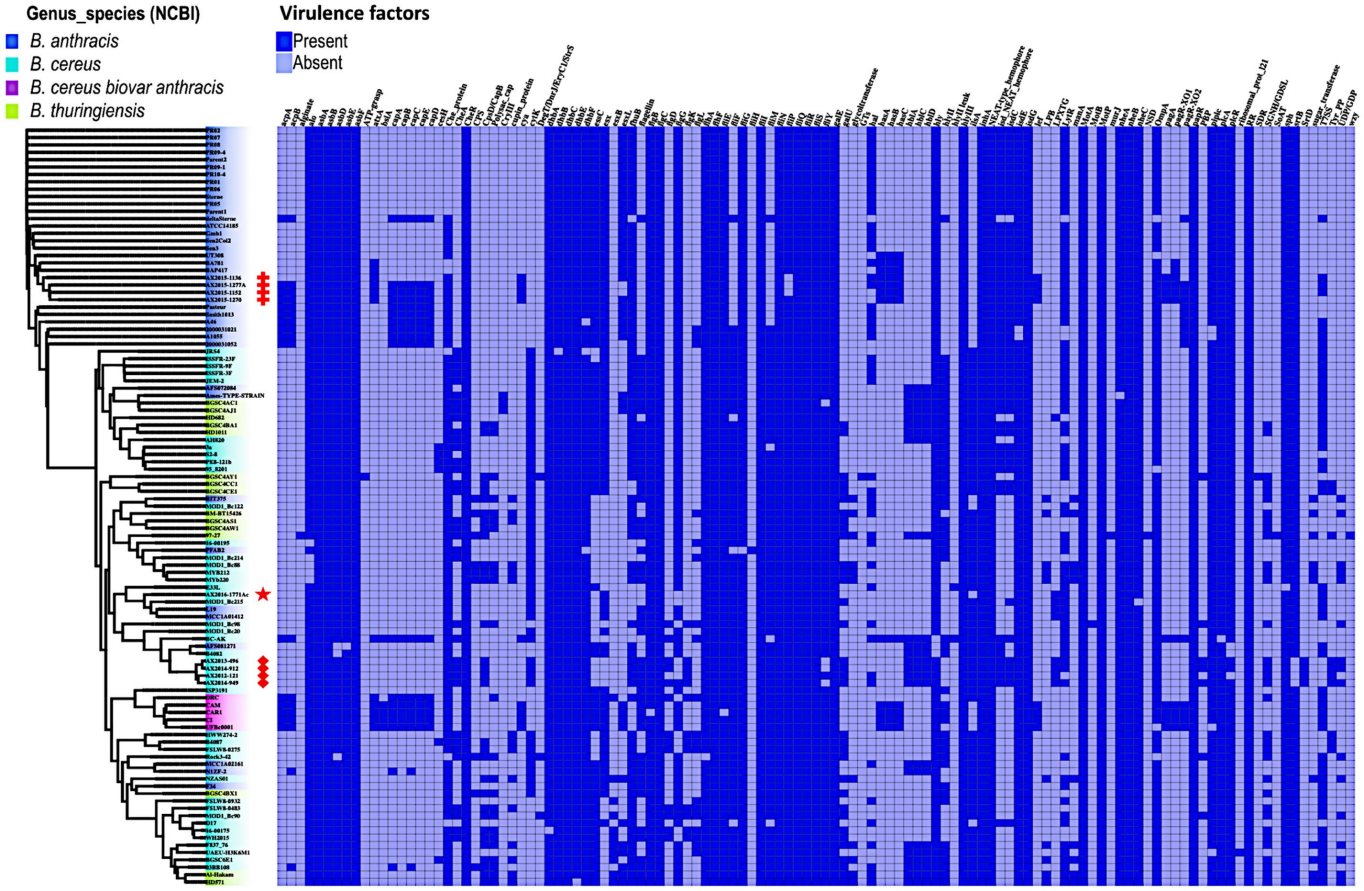
Presence and absence of virulence genes/factors identified in *Bacillus* genomes (*n* = 103) used in this study. The phylogenetic tree is based on the wgSNP of the *Bacillus* genomes. The South African isolates classified as *B. anthracis* (*n* = 4) are marked with red crosses; *B. cereus s. s* (*n* = 4) red diamonds shape, anomalous isolate AX2016-1771Ac marked with the red star.

In this analysis, 117 virulence factor genes were found across 103 genomes analyzed in this study, notably genes which form part of a cluster were counted as individual genes. It has already been established that the 88 GenBank isolates *B. anthracis* (*n* = 36), *B. cereus* (*n* = 37), and *B. thuringiensis* (*n* = 15) do not contain the anthrax virulence genes: edema factor- *cya*, lethal factor- *lef*, the protective antigen -*pagA* located on the pXO1 plasmid (Carroll et al., 2020a,b). In this study, the virulence genes cya, *lef* and *pagA* were found present in the confirmed *B. anthracis* isolates AX2015-1136, AX2015-1152, AX2015-1270, and AX2015-1277A, atypical *B. cereus* BC-AK isolate and the *B. cereus* biovar *anthracis* isolates (UFBc0001, CAR, CAM, and CI). The anomalous isolate AX2016-1771Ac did not contain the anthrax virulence genes *cya*, *lef* and *pagA.* All the capsular genes *cap*-*ABCDE* and the capsule synthesis transcriptional regulator genes *acpA* and *acpB* were detected in the identified and classified traditional *B. anthracis* isolates (deltaSterne, AX2015-1152, AX2015-1270, AX2015-1277A, A1055, 2000031021, 2000013052, A46, Smith1013, and Pasteur), *B. cereus* biovar *anthracis* isolates (UFBc0001, CI, CAM, CAR, and DRC) and the *B. cereus* BC-AK isolate. The *B. cereus* 03BB108 isolated from the environment (dust particles) in the United States and the *B. anthracis* isolate N1ZF-2 from sediments in China both contained the capsule synthesis regulator gene *acpB* and the capsule genes *capA* and *cap*C. The virulence regulator gene of *B. anthracis* (*atxA*), and the *has*-*ACB* gene cluster that encodes for the hyaluronic acid capsule was detected in sequenced *B. anthracis* isolates (AX2015-1136, AX2015-1152, AX2015-1270, AX2015-1277A), and anomalous *B. anthracis* strains BAP417 and BA781, the *B. cereus* BC-AK isolate including the *B. cereus* biovar *anthracis* isolates UFBc0001, CI, CAM, and CAR (Figure 5).

The insecticidal crystalline delta-endotoxin gene *cryIII* was detected in the *B. anthracis* Ames TYPE-STRAIN and *B. thuringiensis* BGSC4AC1 and BGSC4AJ1 strains that shared a common ancestor. At least two or more genes belonging to the gene clusters *asb*-*ABDEF* encoding for the biosynthetic machinery for petrobactin and the *dhb*-*ABCEF* bacillibactin which are siderophores involved in iron acquisition of *Bacillus* species were detected in all the genomes. Although *B. anthracis* is non-motile, gene clusters related to flagellum synthesis (*flg*, *flh*, and *fli*) were present in all the genomes. The non-hemolytic enterotoxin *nhe-ABC* cluster was present in over 99% percent of the genomes. Whereas genes of the hemolytic enterotoxin gene complex *hbl*-*ACD* were detected in 24 genomes which included anomalous *B. cereus*, *B. anthracis*, and *B. thuringiensis*. The cytotoxin gene *cytK* was present in 49.6% of the genomes classified as *B. anthracis*, *B. cereus*, and *B. thuringiensis*, however our sequenced *B. anthracis* strains lack this gene. The hydrolase *cesH* gene which forms part of the cereulide operon was detected in six anomalous *B. cereus* strains (3a, 95_8201, BA087, BGSC6E1, PE8-121b, and S2-8). A more detailed description of the virulence genes is included in Supplementary Table S7.

### 3.7 Determination of the insertion sequences on the 103 genomes

**Figure 6:**
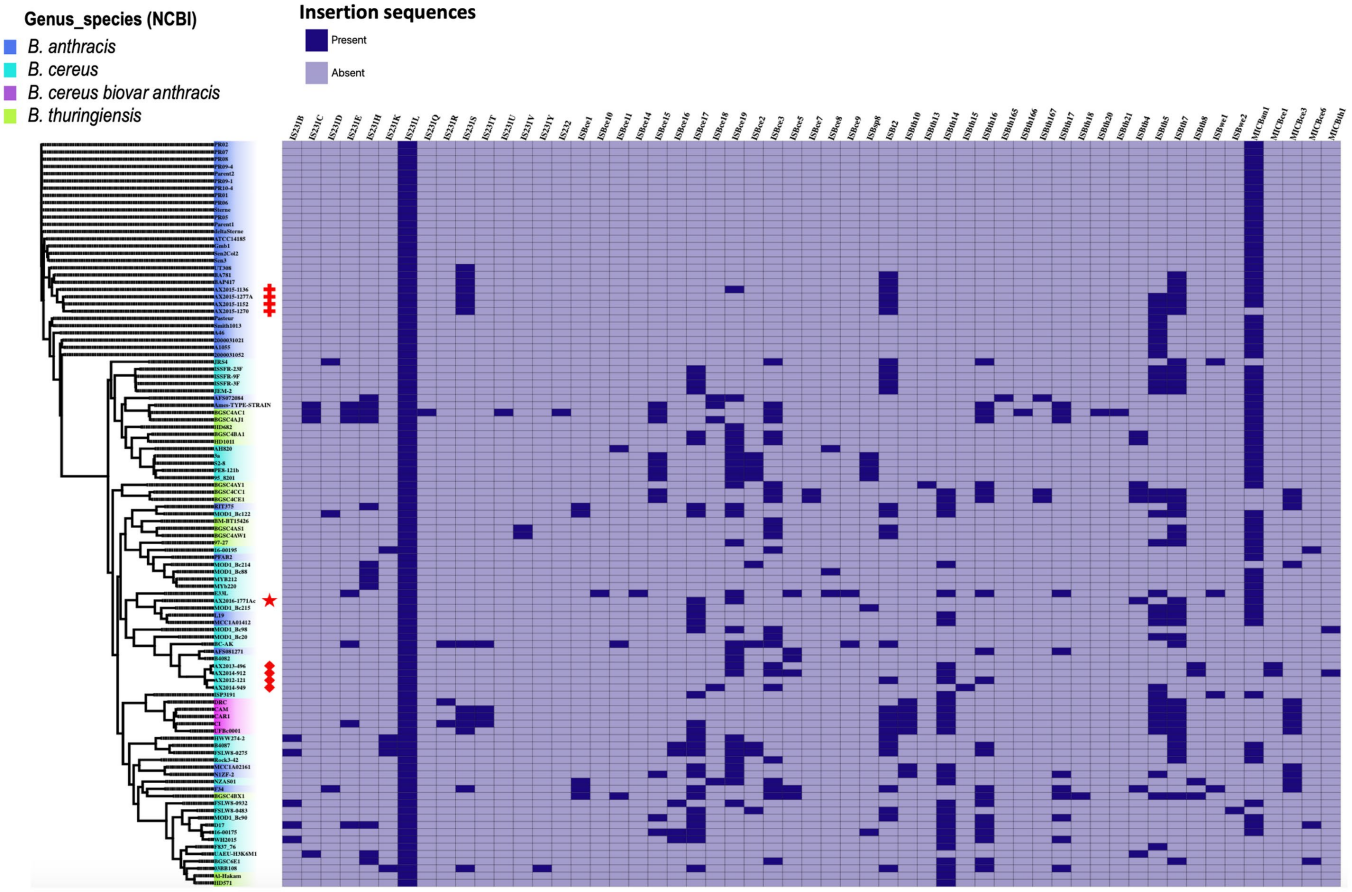
Heat map showing the presence and absence of insertion sequences and mobile insertion cassettes detected on 103 *B. cereus* group genomes analyzed in this study. The phylogenetic tree is based on the wgSNP analysis. The South African isolates classified as *B. anthracis* (*n* = 4) are marked with red crosses; *B. cereus s. s* (*n* = 4) red diamonds shape, anomalous isolate AX2016-1771Ac marked with a red star.

A total of 50 insertion sequences (IS) and five mobile insertion cassettes (MIC) were detected among the 103 genomes (Figure 6). The 50 IS were distributed as follows: IS231 (*n* = 15), ISBce (*n* = 15), ISBth (*n* = 16), ISBwe (*n* = 2), ISBt (1), ISBsp8 (*n* = 1), and IS232 (*n* = 1). Mobile insertion cassettes included MICBan1 (*n* = 1), MICBce (3) and MICBth (*n* = 1). The insertion sequence IS231L was the most prevalent, as it was detected in all 103 genomes analyzed in this study. Among the 30 genomes classified as *B. anthracis* in this study, insertion sequences IS231 (L and S), ISBt (2 and 7) and the insertion cassette MICBan1 were found in AX2015-1136, AX2015-1152, AX2015-1277A, AX1270, and BA781. Other insertion sequences, such as IS2321, ISBwe, and ISBt variants, were less frequent than ISBce and ISBth. The four South African *B. anthracis* isolates contained more IS than the *B. anthracis* isolates in the same cluster (Figure 6). The anomalous *B. cereus* strain AX2016-1771Ac strain sequenced in this study included the insertion sequences IS231L, ISBce (17 and 19), ISBth (4 and 7) and the insertion cassette MICBan1. None of the insertion genes can discriminate typical *B. anthracis* from the anomalous *B. cereus* group strains compared in this study. The *B. anthracis* Ames TYPE-STRAIN and *B. thuringiensis* BGSC4AJ1 and BGSC4AC1 contained similar insertion sequence profiles, which included IS231 (C, E, H, L), ISBce (3 and 15), ISbth (16 and 17), and MICBan1.

## 4 Discussion

In this study, a comparative genomics approach was employed to analyze the nine *B. cereus* group isolates (Table 1), from animal blood smears that also included previously reported four *B. anthracis* strains from anthrax surveillance in KNP (Magome et al., 2024). Pan-genomic and wgSNP analysis was conducted to investigate previously reported “anomalous” *B. cereus* group strains (Liu et al., 2015; Carroll et al., 2020a,b), as well as newly sequenced isolates displaying typical *B. anthracis* and *B. cereus* phenotypes. The study highlights and supports some of the approaches used in the proposed framework by Carroll et al. (2020a,b). We further indicate through a restrictive ANI value of 99% and wgSNP analysis traditional *B. anthracis* can be distinguished from other closely related species which form part of the mosaicus group. Additionally, mobile genetic elements, including insertion sequences, virulence and antibiotic resistance genes across the genomes were investigated to contribute to the growing body isolates that form part of the *B. cereus* group.

The isolates in this study were initially screened for anthrax virulence markers (*pagA*, *lef* and *capB*) and the chromosomal marker Ba-1, with phenotypic characteristics of the isolated recorded as detailed in Ochai et al. (2024). An anomalous strain (AX2016-1771Ac) isolated from *Equus quagga* (Zebra) was discovered when it grouped phylogenetically with *B. anthracis* isolates Ochai et al. (2024). Notably, the strain was initially classified as *B. cereus* using classical microbiological tests, presenting as hemolytic, motile, and characterized as Gram-positive rods with short chains. Furthermore, together with other *Bacillus* isolates (Ochai et al., 2024), five *B. cereus* isolates sequenced in this study presented with positive qPCR detection for the anthrax virulence markers *lef* and/or *pagA* and/or chromosomal marker Ba-1 (Supplementary Table S1). The findings warranted further genomic investigations alongside reference atypical strains *B. cereus* and *B. cereus* biovar *anthracis*, which have reportedly caused anthrax-like diseases in humans and animals (Antonation et al., 2016; Baldwin, 2020).

While classical microbiology techniques, such as culturing and microscopy are still essential for bacterial identification, particularly in anthrax endemic regions (WHO, 2008; Ochai et al., 2024), molecular diagnostics often reveal discrepancies. The work of Ochai et al. (2024) showed that *Peribacillus*, *Lactobacillus*, and *Priestia* species were positive for anthrax virulence markers (*pagA*, *lef*, and *cap*). As an example, the PCR amplicons of *pagA* from *Peribacillus*, *Lactobacillus*, and *Priestia* isolates were sequenced and the BLASTn identification did not match with the *pagA* of *B. anthracis* (Ochai et al., 2024). Further analysis that involved genomic analysis and BLASTn comparison of the anthrax virulence markers in the *Priestia* and *B. cereus* group genomes revealed that these genes (*pagA*, *lef*, and *capB*) were only present in the four confirmed *B. anthracis* genomes with 99.9–100% alignment (Magome et al., 2024). In this study, several isolates with *B. cereus*-like phenotypes were positive for the anthrax-specific *lef* gene in qPCR assays but did not indicate *lef* gene in the genome after sequencing. The *lef* gene was found in the following isolates, with 99.9–100% in the four *B. anthracis* isolates, *B. cereus* biovar *anthracis* isolates and the atypical *B. cereus* strain. This is to be expected as anthrax virulence factors have been detected in these strains and were implicated in causing the anthrax disease to their host animal (Antonation et al., 2016; Baldwin, 2020). Previous studies have reported on false positives occurrence for anthrax virulence markers in non-*B. anthracis* strains from blood smears in anthrax endemic region in Kruger National Park (Lekota et al., 2016; Lekota et al., 2018). As thus the risk of misidentification in routine diagnostics, which could potentially compromise the reliability of anthrax detection assays, particularly in *B. cereus* group is increased if diagnosis is depended on molecular marker detection using PCR (Carroll et al., 2022a,b,c). In order to overcome the misidentification of anthrax using anthrax virulence genes on qPCR assay, Ochai et al. (2024) proposed using a combination of the PCR anthrax virulence markers including the chromosomal Ba-1 marker for increased sensitivity in detecting *B. anthracis* in environmental samples. Other studies have proposed species-specific chromosomal markers found in only in *B. anthracis* that could assist in rapid and accurate detection of *B. anthracis* especially during anthrax outbreaks (Braun et al., 2021; Zorigt et al., 2024).

Genome sequencing remains a gold standard for accurate taxonomic classification, offering high resolution for species delineation (Carroll et al., 2022a,b,c). However, in the *B. cereus* group, genomic ambiguity persists due to the lack of standardized genomospecies thresholds (Carroll et al., 2022a,b,c). The nomenclatural discrepancies observed within the *B. cereus* group will not likely be resolved within the next few years especially when more of these anomalous strains are identified and could be either be misclassified and/or proposed as a new species in the *B. cereus* group based on the database and methodology applied (Gillis et al., 2024). Among the 36 isolates from GenBank classified as *B. anthracis*, 26 clustered with the four confirmed *B. anthracis*, all sharing an ANI threshold of 99%. This suggests that applying a 99% ANI threshold for classifying *B. anthracis* as initially proposed by Jain et al. (2018), could help distinguish typical *B. anthracis* from other group members. Moreover, this was augmented by wgSNP phylogenetic analysis, placing the 26 *B. anthracis* isolates together with the four confirmed South African *B. anthracis* as a cluster.

The remaining 10 anomalous GenBank genomes (Ames TYPE-STRAIN, AFS072084, PFAB2, L19, MCC1A01412, AFS081271, MCC1A02161, N1ZF-2, RIT375, and F34), classified as *B. anthracis*, diverged from typical *B. anthracis* clusters, raising caution in their usage for studies focused solely on typical *B. anthracis*. For instance, the Ames TYPE-STRAIN (GCA_000007845.1) was classified as *B. anthracis* across the genome identification tools (PubMLST and GTDB-TK) but exhibited high ANI with a *B. thuringiensis* isolate and contained insecticidal genes (*cryIII*), a marker typically associated with *B. thuringiensis*. Such findings underscore the challenges of relying solely on ANI or whole-genome SNP analysis for classification within the *B. cereus* group, as even wgSNP analysis did not resolve this discrepancy for the Ames TYPE-STRAIN including the other anomalous *B. cereus* and *B. thuringiensis* genomes.

The use of GTDB and PubMLST classified the sequenced isolates AX2012-121, AX2013-496, AX2014-912, and AX2014-949 from this study as *B. cereus*, belonging to *panC* group IV of the *B. cereus sensu stricto.* These isolates form part of the traditional *B. cereus* based on phenotypic and genomic traits, presented with an ANI of 98%, matching with *B. cereus* ATCC 14579 (GCA_000008725.1). The anomalous isolate AX2016-1771Ac sequenced in this study grouped together with four anomalous *B. anthracis* strains (L19 and MCC1A01412) and *B. cereus* strains (E33L and MOD1_Bc215). Pan-genome analysis identified 14 unique genes, which were solely found on these five isolates. These included genes involved in the physiological functions and processes such as metabolism, cellular homeostasis and transporter cassettes. The anomalous AX2016-1771A isolate contained 146 unique genes, which included ABC transporter permeases, integrase proteins and spore germination proteins (KA, KC, and KB) including aspartyl-phosphate phosphatase Spo0E family proteins known to act as regulator of sporulation which influences development pathways in bacteria such as *B. subtilis* (Perego, 2001).

According to the proposed nomenclature of Carroll et al. (2020a,b), strains that group closely with *B. anthracis* can be classified under the genomospecies *B. mosaicus* (*panC* group III) or simply use the strain number and report the isolate as a member of the *B. cereus* group (Carroll et al., 2022a,b,c). We endorse this classification, although we proposed that the traditional *B. anthracis* species name must be retained by those species that exhibit phenotypic characteristic of *B. anthracis* supported by 99% ANI reference *B. anthracis* with SNP shared by traditional *B. anthracis.* Thus, the anomalous isolate detected in this study based on phenotypic characteristics and genome analysis taking into consideration the impact the classification of species may have on clinical and environmental settings, the isolate AX2016-1771Ac was submitted and classified as *B. cereus* on GenBank. Further description is provided elucidating that the strain belongs to the *panC* group III of the mosaicus group. Recent studies have also reported on the difficulty of classifying novel isolates, they have however relied on placing the isolates in the eight genomospecies groups (I-VIII) as suggested by Carroll et al. (2020a,b). However, species names for novel isolates remain unresolved (Abdelli et al., 2023).

In this study, we also investigated the antimicrobial resistance (AMR) potential of bacterial isolates within the *B. cereus* groups, focusing on the prevalence of specific antibiotic resistance genes (ARGs) that could potentially affect therapeutic efficacy and inform public health monitoring. Monitoring the presence of these ARGs is critical for understanding the distribution of acquired resistance genes and assessing possible clinical interventions for antibiotic-resistant infections (Kompes et al., 2024). Our findings revealed that beta-lactamase genes, specifically *bla1*, *bla2*, and *BcII*, were the most prevalent ARGs among the *B. cereus* group isolates (Figure 4). These genes have been reported to present with a generally low expression in *B. anthracis* attributed to the mutation in regulatory genes such as the *plcR* (Chen et al., 2003; Materon et al., 2003). Among the 30 typical *B. anthracis* isolates studied, a recurring resistance gene profile was observed, characterized by the presence of *BcII*, *Bla1*, *Bla2*, *FosB2*, and *satA* (Figure 4). Notably, *FosB2*, a gene conferring Fosfomycin resistance, was exclusive to the typical *B. anthracis* isolates, distinguishing them from other *B. cereus* group members. The *satA* gene, a streptothricin N-acetyltransferase gene family, associated with nucleoside antibiotic resistance and typically found in actinomycetes in soil-dwelling actinomycetes, suggests a potential environmental acquisition of resistance (Burckhardt and Escalante-Semerena, 2019). Although these resistance determinants are consistent with previous characterizations of *B. anthracis*, further phenotypic testing would provide additional insights into the practical resistance potential of these isolates (Heine et al., 2024). The *B. cereus* showed presence of resistance genes such as beta-lactamases, rifamycin, fosfomycin, and vancomycin in the South African *B. cereus* strains. *Bacillus cereus s.s* are commonly reported in nosocomial infections in clinical settings, proliferation of ARGs among the isolates in the environment could potentially lead to the formation of superbugs which may pose public health risk if ignored (Kowalska et al., 2024).

The presence of virulence factors further emphasizes the pathogenic potential of these isolates. While typical anthrax-toxin genes (*cya*, *lef*, *pagA*) on the pXO1 plasmid (Turnbull, 2002; Liu et al., 2015; Carroll et al., 2020a,b) are absent in the anomalous strains, the anthrax-toxin genes and associated regulatory elements, i.e., *atxA*, were detected in the typical *B. anthracis* (AX2015-1152, AX2015-1270, and AX2015-1277A), as well as in atypical *B. cereus* BC-AK isolate and *B. cereus* biovar *anthracis* strains (UFBc0001, CAR, CAM, and CI), which are implicated in anthrax related diseases in humans and animals (Ochai et al., 2024; Baldwin, 2020). The presence of the pXO1/pBCXO1 and pXO2/pBCXO1 plasmids suggests a shared virulence mechanism across these isolates. Interestingly, one sequenced *B. anthracis* strain, AX2015-1136, lacked the capsular genes (*cap-ABCDE*, *acpA*, *acpB* genes) despite carrying the tripartite anthrax toxin genes, indicative of the pXO1 plasmid’s retention but pXO2 plasmid loss, conversely anomalous isolates deltaSterne, A1055, 2000031021, 2000013052, A46, Smith1013, and Pasteur contained capsular genes but lacked the tripartite anthrax toxin genes, indicative of the pXO2 plasmid retention and loss of pXO1 plasmid. This phenomenon is reportedly likely due to genetic instability from environmental pressures (Liang et al., 2016).

In atypical *B. cereus* strains, the functional expression of both PGA and hyaluronic acid capsules encoded by *has-ABC* located in the pBCXO1 plasmid, facilitates pathogenicity, with the hyaluronic capsule linked to cause anthrax-like disease (Antonation et al., 2016; Baldwin, 2020). This dual-capacity for capsule expression is unique to the atypical *B. cereus* biovar anthracis and is absent in *B. anthracis*, where capsule synthesis is disrupted by mutations within *hasA* (Okinaka et al., 1999). Therefore, the *B. anthracis* isolates only express the PGA capsule, whereas atypical *B. cereus* isolates and *B. cereus* biovar *anthracis* may express both a hyaluronic acid capsule and PGA capsule required for pathogenicity (Oh et al., 2011).

The diversity in the *Bacillus cereus* group’s virulence and resistance factors is further demonstrated by the detection of the hydrolase *cesH* gene, linked to the emetic cereulide operon (*ces-ABCDPTH*), detected in six anomalous *B. cereus* strains (3a, 95_8201, BA087, BGSC6E1, PE8-121b, and S2-8). Although it is associated with a specific group of emetic *B. cereus* strains, non-emetic isolates have been found to carry *cesH* flanking genes which reportedly are closely related to the anthrax-toxin encoding pXO1 plasmid (Ehling-Schulz et al., 2006). The presence of toxin-related gene clusters (*nhe-ABC*, *hbl-ACD*, and *cytK*), which are the hemolysin BL, non-hemolytic enterotoxin, and cytotoxin K, respectively, found in sequenced *B. cereus* isolates AX2012-121, AX2013-496, AX2014-912, and AX2014-949, signals the potential for diarrheal illness, consistent with prior findings associating these clusters with enteric disease (Schoeni and Wong, 2005; Owusu-Kwarteng et al., 2017). The genomic plasticity within these isolates underscored by the presence of insertion sequences and mobile elements, may facilitate AMR and virulence gene transfer, impacting the adaptability and pathogenic potential of *Bacillus* species (Fayad et al., 2019; Bianco et al., 2023). Recent studies have also identified insertions contributing to clarithromycin resistance, underlining the evolutionary adaptability of *B. anthracis* and related species (Maxson et al., 2024).

## 5 Conclusion

In this study, *B. cereus* group isolates that were isolated from blood smear samples in the Kruger National Park presenting with amplification of anthrax-toxin markers during molecular analysis in Ochai et al. (2024) were investigated and resolved using genome-sequence analysis. Following identification of other anomalous strains, 26 out of 36 *B. anthracis* strains may be accepted as part of traditional *B. anthracis* strains. The above suggestion is based on the comparative genomics analysis applied which involved pan-genome, ANI (≥99%) and wgSNP analysis. The anomalous AX2016-17771Ac isolate from this study showed no SNP related to traditional *B. anthracis* and anthrax virulence genes, therefore the isolate was classified as *B. cereus panC* group III of the mosaicus group. The four newly sequenced isolates AX2012-121, AX2013-496, AX2014-912, and AX2014-949 are classified as *B. cereus sensu stricto* of *panC* group IV. Although we were able to distinguish traditional *B. anthracis* from other closely related strains, the relation between *B. thuringiensis* and *B. cereus* remains to be resolved especially those that group in the mosaicus clade. This study also calls into the reassessment of the *B. cereus* taxonomy committee which oversees the guidelines and monitoring of the submission or classification of species in the *B. cereus* group. Most of the anomalous isolates may have been assigned their species names due to the lack of advanced genomic tools, and/or the database available during analysis which contributed to the naming of the isolates within the *B. cereus* group. Our study further highlights the complex interplay of ARGs, virulence factors, and plasmid mobility within *B. anthracis* and other *B. cereus* group species. This genomic diversity necessitates ongoing surveillance and phenotypic assessment to better understand and mitigate the clinical risks posed by potential pathogens in this *B. cereus* group. The insights provided herein contribute to a growing body of evidence on *Bacillus*-related antimicrobial resistance, with potential implications for both therapeutic approaches and public health policies.

## Data Availability

The datasets presented in this study can be found in online repositories. The names of the repository/repositories and accession number(s) can be found in the article/Supplementary material.

## Footnotes

^1^ https://github.com/tseemann/snippy

^2^ https://pubmlst.org/species-id

^3^ https://nmdc.cn/ipga/

^4^ https://www.chiplot.online/index.html
